# Investigating the treatment shortening potential of a combination of bedaquiline, delamanid and moxifloxacin with and without sutezolid, in a murine tuberculosis model with confirmed drug exposures

**DOI:** 10.1093/jac/dkae266

**Published:** 2024-08-07

**Authors:** Kerstin Walter, Lindsey H M te Brake, Ann-Kathrin Lemm, Michael Hoelscher, Elin M Svensson, Christoph Hölscher, Norbert Heinrich

**Affiliations:** Division of Infection Immunology, Research Center Borstel, Leibniz Lung Center, Borstel, Germany; German Center for Infection Research, Partner Site Hamburg-Lübeck-Borstel-Riems, Borstel, Germany; Department of Pharmacy, Radboud Institute of Health Sciences, Radboud University Medical Center, Nijmegen, The Netherlands; Division of Infection Immunology, Research Center Borstel, Leibniz Lung Center, Borstel, Germany; Institute of Infectious Diseases and Tropical Medicine, University Hospital, LMU Munich, Munich, Germany; German Center for Infection Research, Partner Site Munich, Munich, Germany; Fraunhofer Institute ITMP, Immunology, Infection and Pandemic Research, Munich, Germany; Unit Global Health, Helmholtz Zentrum Munich, German Research Center for Environmental Health (HMGU), Neuherberg, Germany; Department of Pharmacy, Radboud Institute of Health Sciences, Radboud University Medical Center, Nijmegen, The Netherlands; Department of Pharmacy, Uppsala University, Uppsala, Sweden; Division of Infection Immunology, Research Center Borstel, Leibniz Lung Center, Borstel, Germany; German Center for Infection Research, Partner Site Hamburg-Lübeck-Borstel-Riems, Borstel, Germany; Institute of Infectious Diseases and Tropical Medicine, University Hospital, LMU Munich, Munich, Germany; German Center for Infection Research, Partner Site Munich, Munich, Germany; Fraunhofer Institute ITMP, Immunology, Infection and Pandemic Research, Munich, Germany

## Abstract

**Background:**

New and shorter regimens against multi-drug resistant tuberculosis (TB) remain urgently needed. To inform treatment duration in clinical trials, this study aimed to identify human pharmacokinetic equivalent doses, antimycobacterial and sterilizing activity of a novel regimen, containing bedaquiline, delamanid, moxifloxacin and sutezolid (BDMU), in the standard mouse model (BALB/c) of *Mycobacterium tuberculosis* (Mtb) infection.

**Methods:**

Treatment of mice with B_25_D_0.6_M_200_U_200_, B_25_D_0.6_M_200_, B_25_D_0.6_M_200_(U_200_^3^) or H_10_R_10_Z_150_E_100_ (isoniazid, rifampicin, pyrazinamide, ethambutol, HRZE), started 3 weeks after Mtb infection. Bactericidal activity was assessed after 1, 2, 3 and 4 months of treatment and relapse rates were assessed 3 months after completing treatment durations of 2, 3 and 4 months.

**Results:**

B_25_D_0.6_M_200_U_200_ generated human equivalent exposures in uninfected BALB/c mice. After 1 month of treatment, a higher bactericidal activity was observed for the B_25_D_0.6_M_200_U_200_ and the B_25_D_0.6_M_200_ regimen compared to the standard H_10_R_10_Z_150_E_100_ regimen. Furthermore, 3 months of therapy with both BDM-based regimens resulted in negative lung cultures, whereas all H_10_R_10_Z_150_E_100_ treated mice were still culture positive. After 3 months of therapy 7% and 13% of mice relapsed receiving B_25_D_0.6_M_200_U_200_ and B_25_D_0.6_M_200_, respectively, compared to 40% for H_10_R_10_Z_150_E_100_ treatment showing an increased sterilizing activity of both BDM-based regimens.

**Conclusions:**

BDM-based regimens, with and without sutezolid, have a higher efficacy than the HRZE regimen in the BALB/c model of TB, with some improvement by adding sutezolid. By translating these results to TB patients, this novel BDMU regimen should be able to reduce treatment duration by 25% compared to HRZE therapy.

## Introduction

Multi-drug resistant tuberculosis (MDR-TB) continues to pose a major global public health threat, with global targets increasingly out of reach.^1^ The long treatment duration and treatment-related toxicity result in poor adherence. As a result, drug resistance is becoming more common and will have an increasing contribution to the TB pandemic.^2,3^

Major impediments in MDR-TB control include inadequate drug susceptibility testing capacity, limited access to or delayed MDR-TB treatment, and lengthy and toxic treatments that cured only half of patients in the past.^3^ In 2022, the WHO has recommended the use of regimens composed of bedaquiline (B), pretomanid, linezolid and moxifloxacin (M) if susceptibility was demonstrated.^4^ However, linezolid at a dose of 600 mg, given for 26 weeks as recommended, still resulted in 24% of patients suffering peripheral neuropathy, and 2% suffering myelosuppression.^5^ The PanACEA consortium is testing a regimen with drugs of similar class and effectiveness [e.g. delamanid (D), a nitroimidazole, and sutezolid (U), an oxazolidinone], with sutezolid being a promising drug candidate to avert the linezolid main toxicities, while showing at least equal activity in murine models.^6^ In humans and mice, orally administered sutezolid is transformed into a sulfoxide metabolite which reaches higher exposures than the parent in both species.^6,7^ The metabolite is active against extracellular *Mycobacterium tuberculosis* (Mtb) while the parent mainly kills intracellular bacteria.^8^

The evaluation of novel treatment regimens in TB patients requires a clear understanding of its exposure-response relationships. Pharmacokinetic confirmation of selected optimal doses in relation to their preclinical outcomes may prevent suboptimal exposures in efficacy studies and avoid inaccurate comparisons between regimen potencies in the translation from animals to man. Therefore, this study aimed to identify human pharmacokinetic equivalent doses before assessing antimycobacterial and sterilizing activity of the BDMU regimen in the standard mouse model (BALB/c) of pulmonary TB, to inform treatment duration in a clinical phase II trial.

## Methods

Female, specific pathogen-free BALB/c mice were purchased from Charles River (Sulzfeld, Germany) or Janvier Labs (Le Genest Saint-Isle, France). Mtb-infected mice were maintained in individually ventilated cages (Ebeco, Castrop-Rauxel, Germany) under biosafety level 3 conditions at the Research Center Borstel, Germany. All animal experimentation was in accordance with the German Animal Protection Law and approved by the animal research ethics committee (Schleswig-Holstein, Germany) before getting permission by the Ministry of Energy, Agriculture, Environment, Nature and Digitalization (Kiel, Germany; permit 36-3/19). The health status of mice was monitored regularly, and moribund mice were killed humanely in accordance with the approved protocol.

For oral administration of drugs, isoniazid (H), rifampicin (R), pyrazinamide (Z) and ethambutol (E) were prepared as previously described^9^ and rifampicin was dosed at least 1 hour before the combination of the other drugs. Bedaquiline was formulated in acidified 20% hydroxypropyl-beta-cyclodextrin solution,^10^ while delamanid, moxifloxacin and sutezolid were pestled in a mortar and resuspended in 5% gum arabic solution. Drug stocks were stored at 4°C and mixed for co-administration on the day of application.

Two sets of pharmacokinetic pilot experiments were performed in 78 uninfected BALB/c mice in total to identify human pharmacokinetic equivalent doses. Mice received various BDMU regimens, namely B_25_D_2.5_M_200_U_100_ [bedaquiline (25 mg/kg), delamanid (2.5 mg/kg), moxifloxacin (200 mg/kg), sutezolid (100 mg/kg)] in pilot 1 (*n* = 24 mice) and B_25_D_0.6_M_200_U_200_ [bedaquiline (25 mg/kg), delamanid (0.6 mg/kg), moxifloxacin (200 mg/kg), sutezolid (200 mg/kg)] and B_25_D_2.5_M_200_U_200_ [bedaquiline (25 mg/kg), delamanid (2.5 mg/kg), moxifloxacin (200 mg/kg), sutezolid (200 mg/kg)] or delamanid_2.5_ (2.5 mg/kg) monotherapy in pilot 2 (*n* = 54 mice). Mice received once daily dosing for 5 days via oral gavage, after which three animals were euthanized at each sampling time point.

Concentrations in mouse plasma were analysed by validated high-performance LC-MS/MS assays at Radboudumc (Nijmegen, the Netherlands). A cross-validation was conducted to confirm performance of a human plasma LC-MS/MS method for BALB/c plasma by comparing responses of five quality control concentrations in mouse plasma with the nominal concentration on a human plasma calibration curve.

Pharmacokinetic parameters were assessed using standard noncompartmental methods in Phoenix WinNonlin version 6.4 (Pharsight Corporation). To determine human pharmacokinetic equivalent doses, area under the curve (AUC) from time point 0 to 24 h (AUC_0-24h_) in mice were compared to clinically relevant exposures in humans at steady state, namely AUC within the dosing interval (AUC_0-t_) at (quasi) steady state. In literature, average AUC_0-t_ in TB patients taking sutezolid (1200 mg 1 ×  daily), bedaquiline (200 mg 3 ×  weekly), delamanid (100 mg 2 ×  daily) and moxifloxacin (400 mg 1 ×  daily) was 7.1,^7^ 98, 7.9 and 25 mg/L*h,^7,11–13^ respectively. AUC_0-24h_ in mice was considered non-equivalent when outside the human ∼35%–50% CV range. We considered that protein binding of sutezolid, delamanid, bedaquiline, and moxifloxacin to be comparable in humans versus mice.^14–17^

To investigate the antimycobacterial and sterilizing activity, mice were exposed to a low dose aerosol infection with the Mtb H37Rv strain, as previously described.^18^ Mice were sacrificed the following day to determine the number of colony-forming units (cfu) implanted in the lung, and 3 weeks later to determine the pulmonary bacterial burden at the start of treatment. Therapy was administered once daily by oral gavage 5 days a week. The pulmonary bacterial burden was assessed after 1, 2, 3 and 4 months of treatment by analysing five mice per regimen and time point and using charcoal agar^6^ for cfu plating. Culture-positive relapse was determined by holding cohorts of at least 14 mice for an additional 3 months after completion of treatment durations of 2, 3 and 4 months. The entire lung homogenate was plated onto Middlebrook 7H10 agar supplemented with 10% bovine serum and the detection of one or greater cfu were defined as culture-positive relapse.

## Results

Cross-validation to confirm performance of our LC-MS/MS method for BALB/c plasma gave robust results. The accuracy of sutezolid, bedaquiline, delamanid and moxifloxacin quality control concentrations was between 97% and 101%, 102% and 111%, 87% and 98%, and 104% and 111%, respectively; the within-run CV was between 1.2% and 1.7%, 2.8% and 6.9%, 1.2% and 2.8%, and 2.7% and 4.3%, respectively. The first pilot experiments for human equivalent dose determination in uninfected mice resulted in sutezolid and main metabolite exposures being below the pre-set target, and delamanid exposures being too high (Table 1). After adjusting doses in pilot 2 for those two drugs to B_25_D_0.6_M_200_U_200_, exposures were found to be in the target range (Table 1).

**Table 1.** BDMU human equivalent dose finding in BALB/c mice

Mouse dose (mg/kg)]	Mouse exposure (AUC_0-24h_) (mg/L*h)	Target (AUC_0-24h_) in humans (mg/L*h)	Conclusions
U 100 (Pilot 1)	1.6–5.2	7.1 (1200 mg once daily)^7^	→Lower in BALB/c than in humans
U 200 (Pilot 2)	4.2–12.7	7.1 (1200 mg once daily)^7^	→Comparable
B 25 (Pilot 1 and 2)	17–29	98 for AUC_0-168h_ in humans (200 mg 3x/week)^12^	→Comparable; 4 days × AUC_0-24h _+ 1×AUC_0-72h_ adds up to ∼120 mg/L*h over 1 week
D 2.5 (Pilot 1 and 2)	18–40	7.9 (100 mg twice daily)^13^	→Higher in BALB/c than in humans
D 0.6 (Pilot 2)	4.4–6.6	7.9 (100 mg twice daily)^13^	→Within predefined target (i.e. ∼35%–50% CV around 8 mg/L)
M 200 (Pilot 1)	14–42	25 (400 mg once daily)^11^	→Comparable

U, sutezolid; B, bedaquiline; D, delamanid; M, moxifloxacin.

Values displayed show AUC results from two different runs of dose/exposure finding.

Therapy of Mtb-infected mice started at a pulmonary bacterial burden of 6.59 log_10_ cfu (Table 2). At completion of the first month of treatment, the standard H_10_R_10_Z_150_E_100_ regimen reduced the bacterial load by 2.89 log_10_ cfu, whereas the B_25_D_0.6_M_200_ and B_25_D_0.6_M_200_U_200_ regimen reduced the cfu counts by 4.04 and 4.35 log_10_ cfu, respectively, so that both BDM-based regimens had a significant greater antimycobacterial activity than the H_10_R_10_Z_150_E_100_ group. The addition of sutezolid to the BDM regimen increased the bactericidal activity by 0.3 log_10_ cfu. After 3 months of treatment, BDM-based regimens, with and without sutezolid, showed faster reduction of lung cfus and culture negativity, while in the H_10_R_10_Z_150_E_100_ group, all mice still showed culture growth (Table 2).

**Table 2.** Lung cfu counts assessed during treatment and relapse assessed 3 months after treatment completion

Regimen	cfu/lung (mean log_10_ ±SD)					Proportion (%) relapsing after treatment for		
	D0	1M	2M	3M	4M	2M	3M	4M
untreated	6.59 ± 0.14	ND	ND	ND	ND	ND	ND	ND
HRZE		3.70 ± 0.55	1.63 ± 0.19	<1^a^	0	15/15 (100%)^b^	6/15 (40%)	0/15 (0%)
BDM		2.55 ± 0.41**	1.30 ± 0.21	0	0	15/15 (100%)^b^	2/15 (13%)	0/15 (0%)
BDM(U^3^)	ND	ND	ND	ND	ND^c^	ND	ND	0/14 (0%)
BDMU		2.24 ± 0.53****	1.36 ± 0.14	0	0	13/15 (87%)	1/15 (7%)	0/16 (0%)

Drug doses (mg/kg): isoniazide H 10, rifampicine R 10, pyrazinamide Z 150, ethambutol E 100, bedaquiline B 25, delamanid D 0.6, moxifloxacin M 200, sutezolid U 200, ND not done.

BDM(U^3^): 3 months of BDMU treatment followed by 1 month of BDM treatment because at the time of the design of the experiment, it was anticipated that human dosing of U would have to be stopped after 3 months for regulatory reasons, due to a limitation of preclinical data available. Therefore, this arm was added to assess the effect of such a premature discontinuation of the drug.

^a^Maximal cfu counts/lung: 6–8 cfu.

^b^One mouse of the cohort was euthanized due to moribund status during the follow-up period. Assessment of lung homogenate revealed culture-positive relapse.

^c^Five mice died or were euthanized due to moribund status during the treatment period.

**P = 0.0011 versus HRZE group; ****P<0.0001 versus HRZE group. The log-transformed cfu data were evaluated by a one-way analysis of variance followed by Bonferroni’s *post hoc* test for multiple comparisons.

Relapse rates after 2 months of therapy were 100% for mice receiving H_10_R_10_Z_150_E_100_ or B_25_D_0.6_M_200_ and 87% (13/15) for mice treated with the B_25_D_0.6_M_200_U_200_ regimen (Table 2). After 3 months of therapy, 13% (2/15) of mice relapsed receiving B_25_D_0.6_M_200_ and 7% (1/15) receiving B_25_D_0.6_M_200_U_200_, compared to 40% (6/15) relapsing in the H_10_R_10_Z_150_E_100_ group. Based on the relapse rates observed at these timepoints of therapy, the addition of sutezolid to the regimen seemed to slightly increase the sterilizing activity. After 4 months of therapy, there were no relapses in any treatment group. Stopping sutezolid after 3 months of therapy did not result in a different relapse rate at 4 months (Table 2).

## Discussion

This study aimed to identify human pharmacokinetic equivalent doses, bactericidal and sterilizing activity of the BDMU regimen, and the additive contribution of sutezolid in the BALB/c mouse model of TB. It was noteworthy that we measured a higher exposure to delamanid at a dose of 2.5 mg/kg, compared to other reports, which might be due to single versus multiple dosing or the co-administration of drugs.^19,20^ The subsequent efficacy experiments revealed that BDM plus sutezolid had a better antimycobacterial and sterilizing activity than the HRZE regimen. The contribution of sutezolid to the regimen however seemed to be small, resulting in a slightly lower relapse rate at two time points.

The present study suggests that BDM represents a promising backbone for further validation in advanced mouse models^18^ that in contrast to BALB/c mice develop a human-like TB pathology. It is important to note that no human study so far was designed to assess the contribution of linezolid to a regimen, so this contribution could be equally small and possibly only result in preventing acquisition of resistance, an important event but usually too infrequent to be measured in clinical trials. Limitations of our report are that PK studies were conducted in uninfected mice and efficacy analyses in BALB/c mice that do not reflect the full range of human TB granulomas.

In summary, translating our results from mice to humans, BDMU should be able to shorten treatment by 25%; i.e. from 6 (HRZE) to 4–4.5 months in TB patients.
